# Supplementary material for: Saffron against Neuro-Cognitive Disorders: An Overview of Its Main Bioactive Compounds, Their Metabolic Fate and Potential Mechanisms of Neurological Protection
Source: Nutrients. 2022 Dec 17;14(24):5368. doi: 10.3390/nu14245368 (PMC9781906; doi:10.3390/nu14245368)
Supplement: Supplementary file 1 [file nutrients-14-05368-s001.zip › nutrients-2084237-supplementary.pdf]

Table S1. Effects of saffron on neurological disorders in recent human intervention studies (double-blind randomized controlled parallel trials, RCTs).

Ref.	Study groups (*N*)	Participants disorder	Country, Sex Age (mean ± SD, y)	Treatment, doses and duration (d)	Results			
					Scale variable (measuring instrument)	Main reported results attributed to saffron (or saffron compound) (Sig)	Effect size (% of change saffron *vs* PLA or drug) (Sig)	Other general comments and results
**Depression**								
[17]	PLA (*31*)<br>Saffron (*31*)	Healthy active adults (practise recreational exercise)	Australia, mixed<br>PLA: 41.8±1.9<br>Saffron: 44±2.2	PLA capsules<br>Saffron capsules (28 mg/d)<br>42 d	Total mood score (POMS-A)	↑-7.3 (*p*=0.014)	↓-8% *vs* ↓-11% (NS)	Measurements done every other week. No significant differences observed. No differences between sexes.
					Emotional distress (anxiety and depression) PROMIS-29	↓-3.2 (*p*=0.009)	↓-6% *vs* ↓-5% (NS)	
[18]	PLA (*36*)<br>Crocin (*36*)	Breast cancer patients during chemotherapy	Iran, women<br>>40 (70%)	PLA tablets (cellulose)<br>Crocin tablets (30 mg/d)<br>120 d	Depression score (BDI)		↓-30% *vs* ↑+23% (NI)	Data estimated from bar figure. Increased leukopenia and reduction of hypersensitivity
					Anxiety score (BAI)	↓ -8.0 (*p*=0.001)	↓-40% *vs* ↑+22% (NI)	
[19]	PLA (*30*)<br>Saffron (*30*)	Overweight/ Obesity, T2DM	Iran, mixed<br>PLA: 51.8 ± 10.9<br>Saffron: 50.5 ± 9.8	PLA capsules<br>Saffron capsules (100 mg/d)<br>56 d	Depression score (BDI-II)	↓-5.9 (*p*<0.001)	↓-31% *vs* ↓-2% (*p*<0.001)	Parallel improvement of some CVDs risk biomarkers and of the overall quality of life
[20]	Sertraline (*15*)<br>Saffron (*19*)	Major depressive disorder	Iran, mixed<br>Sertraline: 66.7 ± 5.3<br>Saffron: 64.5 ± 2.6	Sertraline capsules (100 mg/d)<br>Saffron capsules (60 mg/d)<br>14 d, 28 d, 42 d	Depression score (HDRS)	↓ -11.2 (*p*<0.001)	↓-53% *vs* ↓-59%% (NS)	NS differences and similar effect size to the group treated with sertraline (anti-depressive drug inhibitor of serotonin)
[21]	PLA (*25*)<br>Saffron (*27*)	Overweight mild-to-moderate depression	Iran, women<br>PLA: 39.8 ± 9.2<br>Saffron: 37.0 ± 10.3	PLA capsules<br>Saffron capsules (30 mg/d)<br>14 d, 28 d, 56 d, 84 d	Depression score (BDI)	↓-8.4 (84 d) (NI)	↓-38% *vs* ↓-18% (*p*=0.05)	NS changes in body weight, appetite, food craving or food abstinence

[22]	PLA (*28*)<br>Saffron (*28*)	Depressive disorder associated with post-menopausal hot flashes	Iran, women<br>PLA: 55.4 ± 5.5<br>Saffron: 55.7 ± 6.6	PLA capsules<br>Saffron capsules (30 mg/d containing crocin: 1.7-1.8 mg)<br>14 d, 28 d, 42 d	Hot-flashes (HFRDIS)	↓ -51.8 (42 d) (NI)	↓-75% *vs* ↓-49% (*p*=0.001, 42 d)	Saffron is described as an effective treatment against hot-flashes and depression symptoms in menopause women. Significant differences between groups at all-time points
					Depression score (HDRS)	↓ -7.1 (42 d) (NI)	↓-46% *vs* ↓-28% (*p*=0.007, 42 d)	
[23]	PLA (*14*)<br>Saffron (*15*)	Coronary Artery Bypass Grafting related neuropsychiatric conditions	Iran, mixed<br>PLA: 56.6 ± 5.6<br>Saffron: 58.1 ± 4.4	PLA capsules<br>Saffron capsules (30 mg/d containing crocin: 1.7-1.8 mg)<br>84 d	Anxiety score (HADS)	↓ -1.73 (NI)	↓-43% *vs* ↓-21% (NS)	NS differences between the PLA and the saffron treatment
					Depression score (HADS)	↓ -1.20 (NI)	↓-40% *vs* ↓-10% (NS)	
[24]	PLA (*28*)<br>Saffron (*29*)	Moderate-to severe depression related to recovered consumption of methamphetamine and living with HIV/AIDS	Iran, mixed<br>PLA: 32.9 ± 4.2<br>Saffron: 33.1 ± 3.7	PLA capsules<br>Saffron capsules (30 mL/d)<br>56 d	Depression score (BDI)	↓ -8.7 (NI)	↓-29% *vs* ↓-1.1% (*p*<0.001)	Saffron capsules prepared by distillation with ethanol of saffron at high temperature followed by condensation (unclear procedure and unclear dose)
[25]	PLA (*32*)<br>Saffron (*36*)	Mild-to-moderate anxiety or depressive symptoms	Australia, Spain mixed<br>PLA: 13.9 ± 1.5<br>Saffron: 14.1 ± 1.3	PLA tablets<br>Saffron tablets (28 mg/d containing 3.5% lepticrosalides, including safranal and crocin)<br>56 d	Anxiety and depression scores (RCADS, youth self-reports)	↓-2.98 (separation anxiety, 56d) (*p*<0.001)	↓-44% *vs* ↓-21.3% (*p*<0.003)	Other subscales (general anxiety, panic, obsessions, total anxiety, total internalising) (NS). Results were not clearly consistent with those reported by parents using the same questionnaire
						↓-5.92 (social phobia, 56 d) (*p*<0.001)	↓-34% *vs* ↓-18% (*p*<0.023)	
						↓-3.48 (depression, 56 d) (*p*<0.001)	↓-25% *vs* ↓-6.4% (*p*<0.016)	
[26]	Fluoxetine (*32*)<br>Saffron (*32*)	Mild-to-moderate postpartum depression	Iran, women<br>Fluoxetin: 32.1 ± 4.5	Fluoxetin capsules (20 mg/d)<br>Saffron capsules (30 mg/d containing crocin: 1.7-1.8 mg)	Depression score (HDRS)	↓-7.50 (42 d) (NI)	↓-45% *vs* ↓-46% (NS)	HDRS score reduced equally at the three time points in both groups

			Saffron: 29.2 ± 7.7	7 d, 21 d, 42 d				
[27]	Citalopram (*30*)<br>Saffron (*30*)	Mild-to-moderate major depression with anxious distress	Iran, mixed<br>Citalopram: 34.2 ± 10.4<br>Saffron: 37.9 ± 11.6	Citalopram tablets (20 mg/d for 7 d followed by 40 mg/d for 35 d)<br>Saffron tablet (15 mg/d for 7 d followed by 30 mg/d for 35 d; contains crocin: 1.65-1.75 mg), 14 d, 28 d, 42 d	Anxiety score (HAM-A)	↓-12.04 (42 d) ($p<0.001$)	↓-61% *vs* ↓-63% (NS)	Both the drug and saffron acted similarly decreasing the two investigated scores at the three time points.
					Depression score (HAM-D)	↓ - 10.13 (42 d) ($p<0.001$)	↓-59% *vs* ↓-64% (NS)	
[28]	PLA (*30*)<br>Saffron (*30*)	Mild-to-moderate post-partum associated depression	Iran, women<br>PLA: 28.0 ± 7.4<br>Saffron: 28.1 ± 5.3	PLA tablets<br>Saffron tablets (30 mg/d containing crocin: 5 mg, 33% of the product)<br>28 d, 56 d	Depression score (BDI-II)	↓ -11.6 (56 d) ($p=0.001$)	↓-58% *vs* ↓-22% ($p<0.001$)	More significant impact of the saffron. The reduction is also seen at the 28 d of treatment.
[29]	PLA (*38*)<br>Saffron D1 (*42*)<br>Saffron D2 (*41*)	Healthy (self-reporting low mood but no mood or depression disorder diagnosed)	Australia, mixed<br>PLA: 40.4 ± 14.0<br>D1: 40.4 ± 12.7<br>D2: 36.7 ± 14.6	PLA tablets<br>Saffron tablet (D1: 22 mg/d and D2: 28 mg/d standardized to contain 3.5% lepticrosalides: mix bioactives, i.e. crocin isomers, safranal)<br>28 d	Mood score (POMS - TMD)	↓-30.8 (D2 group) (NI)	↓-77% D2 *vs* ↓-14% ($p<0.001$)	General significant improvement in most POMS subscales (tension, depression, confusion, fatigue and vigour). No significant treatment effect with D1
					Negative affects score (PANAS)	↓-6.6 (D2 group) (NI)	↓-32% D2 *vs* ↓-13% ($p=0.001$)	
					Depression score (DASS-21)	↓ -11.2 (D2 group) (NI)	↓-167% D2 *vs* ↓-48% ($p=0.001$)	
[30]	PLA (*17*)<br>Crocin (*16*)	MetS	Iran, mixed<br>PLA: 48 (37, 55)<br>Crocin: 45 (34.5, 50)	PLA tablets<br>Crocin tablets (30 mg/d)<br>56 d	Depression score (BDI)	↓ NI ($p=0.005$)	↓-69% *vs* ↓-30% ($p=0.013$)	Unclear results and unclear association with other general serum biomarkers
[31]	PLA (*30*)<br>Saffron (*30*)	Anxiety and depression	Iran, mixed<br>PLA: 43.6 ± 8.8<br>Saffron: 42.8 ± 10.7	PLA capsules<br>Saffron capsules (100 mg/d)<br>84 d	Depression score (BDI)	↓ -6.7 ($p<0.05$)	ND (PLA ↓-4.4) ($p<0.05$)	No information on the baseline scoring for BDI and BAI
					Anxiety score (BAI)	↓ -8.7 ($p<0.05$)	ND (PLA -5.5) ($p<0.001$)	
**Sleeping disorders**								

[17]	PLA (*31*)<br>Saffron (*31*)	Healthy active adults (practise recreational exercise)	Australia, mixed<br>PLA: 41.8±1.9<br>Saffron: 44±2.2	PLA capsules<br>Saffron capsules (28 mg/d)<br>42 d	Total sleep time	↓-0.16<br>(*p*=0.183)	↓-2.3% *vs* ↓-0.9%<br>(*p*=0.598)	Measurements done every other week. No significant differences observed. No differences between sexes.
					Sleep efficiency (%)	↓-0.33<br>(*p*=0.475)	↓-0.4% *vs* ↓-0.01%<br>(*p*=0.170)	
[32]	PLA (*36*)<br>Saffron (*34*)	Healthy active adults with self-reported unsatisfactory sleep	Australia, mixed<br>PLA: 52.2±1.9<br>Saffron: 55.0±0.9	PLA capsules<br>Saffron capsules (28 mg/d)<br>28 d	Sleep quality rating	↑+0.59<br>(NI)	↑22% *vs* ↑8.4%<br>(*p*=0.023)	Several other questionnaires did not show significant effects. There were no significant differences between doses (28 mg *vs* 14 mg)
					Mood rating after waking	↑+0.45<br>(NI)	↑14% *vs* ↓1.6%<br>(*p*=0.009)	
					ISQ	↓-5.7<br>(*p*<0.001)	↓17% *vs* ↓4%<br>(*p*=0.013)	
[19]	PLA (*30*)<br>Saffron (*30*)	Overweight/ Obese T2DM	Iran, mixed<br>PLA: 51.8 ± 10.9<br>Saffron: 50.5 ± 9.8	PLA capsules<br>Saffron capsules (100 mg/d)<br>56 d	Sleep quality (PSQI-Global)	↓-4.7<br>(NI)	↓+57% *vs* ↓-16%<br>(*p*<0.001)	Some PSQI subcomponents improved, others not affected. Sexual desire not affected
					Life quality (DQOL-BCI)	↑+8.5<br>(*p*<0.001)	↑+21% *vs* ↑+1%<br>(*p*<0.001)	
[33]	PLA (*30*)<br>Saffron (*29*)	Mild-to-moderate chronic primary sleep disorder; mild-to-moderate anxiety	Belgium, mixed<br>PLA:<br>44.0 ± 15.0<br>Saffron:<br>46.0 ± 13.0	PLA capsules<br>Saffron capsules (15.5 mg/d containing ≈0.9 mg crocin, 0.7 mg safranal)<br>42 d	Sleep quality (TIB - actigraphy)	↑+14.1 (min)<br>(NI)	↑+3% *vs* ↓-1.8%<br>(*p*<0.05)	Other five sleep subcomponents showed NS changes
					Sleep quality (LSEQ)	No clear effect reported	ND	LSEQ: None of the four scores were significantly different between groups
					Sleep duration score' (PSQI)	↓-0.41<br>(*p*<0.05)	↓-37% *vs* ↑+8.7%<br>(*p*<0.05)	PSQI: Other six scores and global score were NS different between groups
					Life quality (SF-36: Social function' score)	↑+3.1<br>(NS)	↑+4% *vs* ↑+17%<br>(*p*<0.05)	Some improvement in other scores seen in the saffron group but differences with the PLA group were all NS
[34]	(Crossover)<br>PLA (*24*)<br>Crocetin (*24*)	Mild sleep complains	Japan, mixed<br>50.8 ± 6.9	PLA capsules<br>Crocetin capsules (7.5 mg/d)<br>14 d	Sleep quality (EEG, delta power)	↑+198 (μV^2/min)<br>(NI)	↑+9% *vs* ↓+7%<br>(*p*=0.038)	Other five sleep parameters were NS
					Sleep quality (OSA-MA)	↑+3.6 (Sleepiness on rising) (NI)	↑+25% *vs* ↑+10%<br>(*p*=0.011)	Other three scores were all NS

						↑+2.1 (feeling refreshed) (NI)	↑+12% *vs* ↑+0.6% (p=0.007)	
[29]	PLA (*38*)<br>Saffron D1 (*41*)<br>Saffron D2 (*42*)	Healthy (self-reporting low mood but no mood or depression disorder diagnosed)	Australia, mixed<br>PLA: 40.4 ± 14.0<br>D1: 40.4 ± 12.7<br>D2: 36.7 ± 14.6	PLA<br>Saffron tablet (D1: 28 mg/d and D2: 22 mg/d standardized to contain 3.5% lepticrosalides: mix bioactives, i.e. crocin isomers, safranal)<br>28 d	Sleep quality (PSQI)	No significant improvement	ND	Poor presentation of these results. Absence of data or Figure
**Cognitive disorders**								
[35]	Methylphenidate (*25*)<br>Saffron (*25*)	Attention deficit/ hyperactivity disorder	Iran, mixed<br>MPH: 9.1 ± 2.2<br>Saffron: 8.3 ± 1.6	Methylphenidate (5 to 30 mg/d increasing dose per week)<br>Saffron (20 to 30 mg/d)<br>21 d, 42 d	Attention/ Hyperactivity score (Teacher/Parent ADHD)	↓ -23.7 (parents total score) (p<0.001)<br>↓ -19.6 (teachers total score) (p<0.001)	↓+69% *vs* ↓-69% (NS)<br>↓+57% *vs* ↓-57% (NS)	Both the parents and the teachers rating show similar results
[23]	PLA (*19*)<br>Saffron (*18*)	Coronary artery bypass grafting related neuropsychiatric conditions	Iran, mixed<br>PLA: 56.6 ± 5.6<br>Saffron: 58.1 ± 4.4	PLA capsules<br>Saffron capsules (30 mg/d containing crocin: 1.7-1.8 mg)<br>84 d	Memory score (WMS-R)	↑ +4.53 (NI)	↑+6.6% *vs* ↑+6.6% (NS)	NS differences between the PLA and the saffron treatment
					Mental status score (MMSE)	↑ +0.78 (NI)	↑+2.7% *vs* ↑+1.3% (NS)	
[36]	(single-blind)<br>Control (*18*)<br>Saffron (*17*)	Mild cognitive impairment	Greece, mixed<br>Control: 69.7 ± 7.3<br>Saffron: 71.5 ± 6.7	No information on the dose used (?)<br>365 d	Mental status score (MMSE)	↑ +0.77 (NI)	↑+3% *vs* ↓-3% (p=0.015)	Other various psychometric tests were not significantly modified. Some improvement in brain images with MRI (n=4) and EEG recording (n=3)
[37]	Memantine (*30*)<br>Saffron (*30*)	Moderate to severe Alzheimer's disease	Iran, mixed<br>Memantine: 77.5 ± 7.9<br>Saffron: 77.7 ± 8.1	Memantine (20 mg/d)<br>Saffron capsules (30 mg/d containing crocin: 1.7-1.8 mg crocin)<br>365 d	Cognitive impairment score (SCIRS)	↓ -1.9 (NI)	↓-9.2% *vs* ↓-7.8% (NS)	No improvement of cognitive function (*). No significant difference between groups for the MMSE and FAST scores

[38]	PLA (*20*)<br>Saffron (*22*)	Moderate to severe Alzheimer's disease	Iran, mixed<br>PLA: 73.1 ± 4,7<br>Saffron: 72.7 ± 3.9	PLA capsules<br>Saffron capsules (30 mg/d containing 0.13-0.15 mg safranal, 1.7-1.8 mg crocin)<br>112 d	Cognitive impairment score (ADAS-cog)	↓ -3.7<br>(NI)	↓-15% *vs* ↑+16%<br>(*p*<0.0001)	Short term effect of saffron against AD cognitive impairment
					Dementia score (CDR-SB)	↓ -0.67<br>(NI)	↓-10% *vs* ↑+9.8%<br>(*p*<0.0001)	

N: Final participants per group; PLA: Placebo; Sig: results that were reported to be significantly different in the changes from baseline between groups are indicated; NS: Not significant: NI: Not indicated; ND: Not determined; ADAS-cog: Alzheimer disease assessment scale – cognitive subscale (subjects with AD have significantly higher scores, the lower, the better); ADHD: Attention deficit and hyperactivity disorder (the higher, the worse); BAI: Beck anxiety inventory (higher score, higher anxiety; the lower, the better); BDI: Beck depression inventory (the higher, more depression; the lower, the better) (II: Second edition); CDR-SB: Clinical dementia rating scale – sum of boxes (the higher, the more severe dementia, the lower, the better); DASS: Depression Anxiety Stress Scales (a higher score, greater severity); Delta power: primary efficacy endpoint (the higher, the better); DQOL-BCI: Diabetes-specific Quality-of-Life brief clinical inventory (lower score is poorer QoL); EEG: Electroencephalogram; FAST: Functional assessment staging (the higher stage, the worse); HADS: Hospital Anxiety and Depression Scale (the higher, the worse); HAM-A: Hamilton Rating Scale for Anxiety (the lower, the better); HAM-D: Hamilton Rating Scale for Depression (the lower, the better); HDRS: Hamilton depression rating score (the lower, the better); HFRDIS: Hot flashes-related daily interference scale (the higher, the worse); ISQ: Insomnia symptoms questionnaire; LSEQ: Leeds sleep evaluation questionnaire (the higher, the better); MMSE: Mini Mental Status Examination (the lower, the worse); MRI: Magnetic resonance imaging; OSA-MA: Oguri-Shirakawa-Azumi sleep inventory middle age and aged version questionnaire (standardized for idle-age and elderly Japanese people; the higher, the better); PANAS: Positive and Negative Affect Schedule (the lower, the better); POMS: Profile of mood states; PROMIS: Patient-reported outcomes measurement information; PSQI: Pittsburgh sleep quality index (the lower, the better); SCIRS: Severe Cognitive Impairment Rating scale (Higher score, greater impairment; the lower, the better); SF-36: short-form 36 items life quality questionnaire (the higher, the better); TIB: Time in bed (minutes); TMD: Total mood disturbance (higher scores indicating increased mood disturbance); WMS-R: Wechsler Memory Scale—Revised (the higher, the better).
